# Supplementary material for: Presence and Persistence of Andes Virus RNA in Human Semen
Source: Viruses. 2023 Nov 17;15(11):2266. doi: 10.3390/v15112266 (PMC10675069; doi:10.3390/v15112266)
Supplement: Supplementary file 1 [file viruses-15-02266-s001.zip › viruses-2688931-supplementary.pdf]

Supporting information S1 – virus isolation protocols

ANDV isolation was attempted from various samples taken at different time points. Isolation was not successful for any of the samples or culture systems used.

Sample collection and selection of samples for virus isolation experiments: Semen samples were collected in sterile containers. Samples for virus isolation were selected to have high viral loads as defined by qRT-PCR (2017-V-004 semen sample, 40 days after onset of symptoms; 2017-V-022, semen sample, 82 days after onset of symptoms; 2017-V-096, semen sample, 320 days after onset of symptoms).

Sample processing and storage: Sample 2017-V-004 was used for infection of Vero E6 cells directly upon arrival in the laboratory, i.e. without being frozen. Infection experiments using BSR/Vero cell cultures, primary human epithelial cell cultures and 3D human airway epithelia cultures were done at a later time point, wherefore the respective samples (2017-V-022, 2017-V-096) were stored at -80°C. In addition to the use of original sample material, a homogenization protocol was applied to the samples: 400 µl of sample were suspended in 400 µl of PBS and centrifuged for 5 min. at 600 × g. Supernatant was discarded, and the pellet was washed with 1 ml of PBS. Cells were pelleted again by a centrifugation step of 5 min. at 600 × g. Washing was repeated one more time, then the pellet was suspended in 300 µl of PBS. Thereof, 200 µl were added to a tube containing ceramic beads with 1.4 mm diameter (CK14 tubes, Precelly, Bertin Technologies, Montigny-le-Bretonneux, France). The samples were homogenized at 5,000 × rpm twice for 25 sec, with a 10 sec. break. The tubes were centrifuged for 1 min. at 10,000 × g,

and the supernatant was short time stored at 4°C prior to being used in infection experiments as described below.

Vero E6 cell cultures:

Vero E6 cells were obtained from the American Type Culture Collection (Institut Pasteur, ATCC CRL-1586). Cells were maintained in MEM (Minimal Essential Medium) supplemented with 10 % fetal calf serum (FCS), 1.25 % L-glutamine and 0.5 % non-essential amino acids (NEAA) (Biochrom, Berlin, Germany) in 150 cm^2 Corning culture flasks (Faust, Schaffhausen, Switzerland) at 37°C without CO_2. The day before infection, cells were seeded into a 25 cm^2 TBP culture flask (Faust). Thirty min prior to infection, the cell culture medium was exchanged with 2.5 ml of MEM supplemented as described above but using only 2 % FCS. Cultures were infected with 100 µl of sample and absorption was allowed for 1 h at 37°C without CO_2. Ten ml MEM supplemented with 2 % FCS were added, and flasks were incubated for 10 days at 37°C without CO_2. On day 10, samples from all cell culture supernatants were taken for qRT-PCR analyses as described below. Then, cell culture medium was discarded, and cells were detached using 2 ml trypsin (Biochrom). Eight ml MEM supplemented with 2 % FCS were added and cells were resuspended. One ml of the cell suspension was passaged to freshly prepared cell cultures (i.e. passage 1). Incubation, sampling and passaging were repeated six times. Virus became undetectable by qRT-PCR after the first passage and remained undetectable throughout the five subsequent passages.

BSR/Vero cell cultures:

BSR (baby hamster kidney cells transfected with T7) and Vero cells were obtained from the institute of microbiology IMUL in Lausanne, Prof. Stefan Kunz and the American Type Culture Collection (ATCC CCL-81), respectively. They were mixed 1:1 and cultured in DMEM (Dulbecco's Modified Eagle Medium) supplemented with 10 % FCS, 1 % penicillin-streptomycin and 1.25 % L-glutamine, 1 % NEAA (Biochrom) and 1 mg/ml Geneticin G418 (Promega, Madison, US) in 150 cm^2 Corning culture flasks (Faust) at 37°C without CO_2. The day before infection, cells were seeded into a 25 cm^2 TPB culture flask (Faust). Thirty min prior to infection, the cell culture medium was exchanged with a) 2.5 ml of DMEM supplemented as described above but using only 2 % FCS or b) 2.5 ml of DMEM supplemented with 4 % PEG 8,000 (Promega), 2 % DMSO and 2 % FCS. Cultures were infected with 100 µl of sample and absorption was allowed for 1 h at 37°C without CO_2. 10 ml DMEM supplemented as described above (a) or b) were added, and flasks were incubated for 10 days at 37°C without CO_2. On day 10, samples from all cell culture supernatants were taken for qRT-PCR analyses as described below. Then, cells were scraped and resuspended in 2 ml of culture medium, and the cell suspension was used to infect freshly prepared cell cultures (i.e. passage 1). Also, an aliquot of the cell suspension was used for qRT-PCR analysis. Incubation, sampling and passaging were repeated a total of three times. Virus became undetectable by qRT-PCR after the first passage and remained undetectable throughout the two subsequent passages.

Primary human epithelial cell cultures hAECB and hAECN:

Primary human epithelial cell cultures were obtained from Epithelix (Geneva, Switzerland). The cells were isolated from bronchial (hAECB) and nasal (hAECN) biopsies by the manufacturer and contain basal, goblet, and ciliated cells. Individual

aliquots were thawed and passaged for a maximum of three passages. Cells were maintained in hAEC culture medium (Epithelix) in 75 cm^2 Corning culture flasks at 37°C and 5 % CO_2. The day before infection, cells were seeded into a 25 cm^2 TPB culture flask (Faust). Thirty min prior to infection, the hAEC culture medium was exchanged with 2.5 ml of fresh medium. Flasks were infected with 100 µl of sample and absorption was allowed for 1 h at 37°C and 5 % CO_2. 10 ml hAEC culture medium were added, and flasks were incubated for 10 days at 37°C with 5 % CO_2 and 99 % relative humidity. Culture medium had to be renewed every three to four days; at each medium renewal, culture medium samples were taken for qPCR analyses as described below. After ten days, cells were detached by adding 1.3 ml of trypsin (Biochrom) to each flask and incubating for 10 min at room temperature. Then, cells were resuspended in a Falcon tube in 10 ml hAEC culture medium and pelleted by centrifuging the tubes for 10 min. at 2,000 × g. The pellet was resuspended in 1 ml hAEC medium and used to infect freshly prepared cell cultures (i.e. passage 1). Also, an aliquot of the cell suspension was used for qRT-PCR analysis. Incubation, sampling and passaging were repeated a total of three times. Virus became undetectable by qRT-PCR after the first medium renewal (i.e. at day three post infection) and remained undetectable throughout the two subsequent passages.

Three-dimensional human airway epithelia (MucilAir™): Three-dimensional human airway epithelia were obtained from Epithelix (Geneva). The so-called MucilAir™ were reconstituted from human primary cells and delivered by the manufacturer as fully differentiated and functional three-dimensional cultures on semiporous inserts. The inserts were maintained in 24-well plates on MucilAir culture

medium (Epithelix), and basolateral medium was exchanged every three days. In addition, the cell layer was washed with 200 µl Hank's balanced salt solution (HBSS, Gibco; Thermo Fisher Scientific, Darmstadt, Germany) twice a week. Cultures were incubated at 37°C and 5 % CO_2 for 14 – 21 days prior to infection experiments. For infection experiments, 100 µl of sample were suspended in 500 µl of MucilAir culture medium medium. One hundred µl were added in droplets on top of the cultures, and 500 µl were added to the bottom of the well. After 1 h of incubation at 37°C and 5 % CO_2, the inoculum from the apical side was transferred to the basal side of the well, and plates were incubated at 37°C and 5 % CO_2. On day three post infection, medium was exchanged, and the cell layer was washed with 200 µl of HBSS. Samples from the culture medium and the washing solution were taken for qRT-PCR analysis. Medium changing, cell layer washing and sampling for qRT-PCR were repeated another seven times in intervals of three to four days, resulting a total incubation of 30 days. Virus became undetectable by qRT-PCR after the first medium change for all samples.

Monitoring of viral growth using qRT-PCR:

One hundred µl of sample were inactivated in 400 µl of AVL buffer (Qiagen). Thereof, 400 µl were used for nucleic acid extraction using the EZ1 Advanced XL system, the EZ1 Advance XL Virus Card v2.0 and the EZ1 Virus Mini Kit v2.0, with an elution volume of 150 µl. qRT-PCR was then carried out as described in (1).

References

1. Kuenzli AB, Marschall J, Schefold JC, Schafer M, Engler OB, Ackermann-Gaumann R, et al. Hantavirus Cardiopulmonary Syndrome Due to Imported Andes Hantavirus Infection in Switzerland: A Multidisciplinary Challenge, Two Cases and a Literature Review. Clin Infect Dis. 2018;67(11):1788-95.
